# Understanding the structure of a country’s health service providers for defence health engagement

**DOI:** 10.1136/bmjmilitary-2020-001502

**Published:** 2020-06-04

**Authors:** Martin Bricknell, S Hinrichs-Krapels, S Ismail, R Sullivan

**Affiliations:** 1 Conflict and Health Research Group, King's College London⁠—Strand Campus, London, UK; 2 Policy Institute, King's College London, London, UK; 3 Department of Primary Care and Public Health, Imperial College London Faculty of Medicine, London, UK; 4 Conflict and Health Research Group, King's College London, London, UK

**Keywords:** public health, medical education & training, organisation of health services

## Abstract

There are a variety of structural and systems frameworks for describing the building blocks of country’s public health and health systems. In this paper, we propose a conceptual framework for a holistic view of a country’s health service providers in order to inform the plan for Defence Health Engagement activities with partner countries. This includes all potential government ministries involved in healthcare provision, the independent, private sector and the non-government organisation/charity sector. The framework provides a visualisation to support the analysis of a country’s health services providers. We propose that recognising and analysing the different contributions of all these national health providers is essential for understanding the wider political economy of a nation’s health systems. This can inform a plan of Defence Health Engagement for capacity building in crisis response, development and health systems strengthening.

## Introduction

The role of the Defence Medical Services in Defence Engagement was formalised by the creation of the Centre for Defence Healthcare Engagement in 2015.^1^ Defence Healthcare Engagement (DHE) requires a full understanding of the context of the partner country, including the role of the military health system within the wider health economy.^2^ This paper provides a conceptual framework to support the development of tools to support practitioners to undertake such analyses.

The terms ‘*health system*’, ‘*health sector*’ or ‘*health economy*’ are used interchangeably to describe the range of stakeholders and actors within a state that provide health services for a country’s population.^3^ WHO defines a health system as *consisting of all organisations, people and actions whose primary intent is to promote, restore or maintain health*.^4^ The definition is supported by a conceptual framework for health systems based on the following thematic building blocks: service delivery; health workforce; information; medical products, vaccines and technologies; financing; leadership and governance (stewardship). There is a need to deconstruct the service delivery building block in order to understand the range of providers that meet the health needs of the country’s population and to ensure deconfliction with DHE activities.

While a government’s Ministry of Health (or, Ministry of Public Health) will have primary responsibility for the technical aspects of stewardship for a nation’s health sector, it will not be the only actor or the only provider of health services. The WHO defines health services as ‘*service delivery systems that are responsible for providing health services for patients, persons, families, communities and populations in general, and not only care for patients’.* 5 In general terms, health services providers may be divided into horizontal services that are designed to provide comprehensive coverage of a populations’ health needs (such as those financed by public health systems, eg, primary care) or vertical services that are designed to provide coordinated interventions for a specific condition (such as HIV/AIDS).^6^

This paper substantially refines the framework based on experiences from Op HERRICK, partially described in a paper published in 2011^7^ and issued as policy in Joint Doctrine Note 3/14 The Military Medical Contribution to Security and Stabilisation (8, withdrawn).^8^ The changes result from testing the framework and explanation in military and civilian educational settings since the idea was first conceived. This paper also provides a full explanation of the framework and introduces new ideas such as ‘*security ministries*’ and ‘*independent health services*’. It expands the frame of reference to capture all the actors that may be involved in providing health services for a country. Beyond Ministries of Health, this framework explicitly includes the potential roles of wider actors such as other government ministries (Ministries of Higher Education, Defence, Interior), the independent sector and International Agencies (IAs)/non-Government Organisations (NGOs)/charities. The framework is offered as an analytical tool to identify all providers within a country’s health system to support comparisons of providers within and between countries.

## The components of ‘*a country’s health service providers*’

### Overview

**Figure 1:**
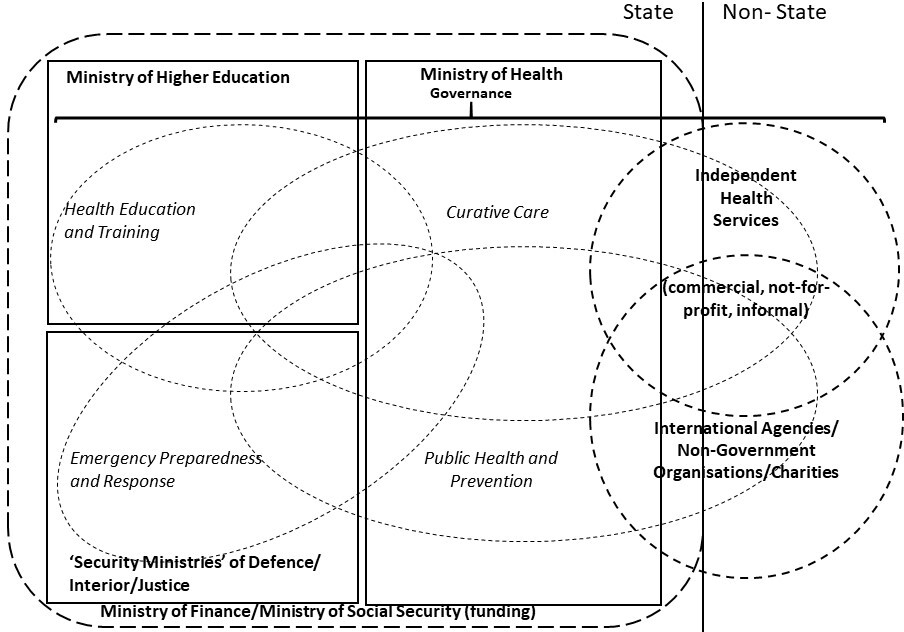
A country’s health service providers framework.

‘*A country’s health service providers*’ framework that describes all potential providers of health services within a country is shown in figure 1. Broadly, these are divided into state and non-state services. In principle, state services are funded by the Ministry of Finance through budget allocations to government ministries based on political choices using government income from taxation. In some countries, compulsory contributions to social security funds may be managed separately from taxation income and so the Ministry of Social Security is also shown as a source of government funding. External donors may also provide direct grant support to government ministries, and non-government third-party funders (including non-public insurers) may also provide finance to government-provided hospitals. The model shows five government ministries that potentially provide health services: Ministry of Health, Ministry of Higher Education, Ministry of Defence, Ministry of Interior and Ministry of Justice. There could be even more in individual countries.

Non-state providers lie outside formal control of the government, although should be subject to national laws and regulations. These are independent providers (commercial providers, not-for-profit providers and the informal sector including pharmacists and traditional healers) and the IA/NGO/charity sector. The Ministry of Health is often the focus for stewardship of the health system including the frameworks for regulation of healthcare workers, pharmaceuticals and medical devices. The relative size of each sector is stylised in figure 1. The size will vary from country to country and on the unit of measure (eg, dependent population, proportion of nation’s health expenditure, per capita expenditure). Each of the sectors will be considered in turn.

The model also shows four types of health services: curative care, public health and prevention, emergency preparedness and response and health education and training. These types overlap between providers, although the principle focus for policy and regulation is likely to lie with a specific government department.

### The Ministry of Health

The essential functions/operations of the public health system managed by Ministries of Health include: surveillance of population health and well-being; monitoring and response to health hazards and emergencies; health protection; health promotion; disease prevention; assuring governance for health and well-being; assuring a competent and proficient public health workforce; assuring sustainable organisational structures and financing; advocacy communication and social mobilisation for health; advancing public health research to inform policy and practice.^9 10^ This list reflects an assumption that governance for population health lies at the central state level (referred to as ‘*public health and prevention*’ in figure 1).

Many states devolve the management of government funds for the delivery of healthcare for patients to regional or local bodies (‘*curative care*’ in figure 1). Devolved governance may have local democratic accountability or may be a delegated responsibility of the central government Ministry of Health depending on the legal, political and sociocultural construct. It may even lie outside state control in areas of contested governance. This will also be influenced by the funding model that will be a balance between central taxation, local taxation, insurance and out-of-pocket expenditure. The division of responsibilities between central and local governance will also depend on the structure of individual clinical services.

### The Ministry of Higher Education

The capability and capacity of the healthcare workforce is one of the biggest strategic challenges facing all health systems.^11^ Responsibility for training the workforce may lie with the Ministry of Higher Education rather than the Ministry of Health, if responsibility for healthcare education aligns with other undergraduate/postgraduate education programmes within universities. The setting for the education of healthcare professionals is often shared between university teaching environments and clinical settings such as teaching hospitals. These teaching hospitals may be run by the Ministry of Higher Education and therefore operate within the state health sector but not subordinate to the Ministry of Health.

### The ‘*Security Ministries*’—Ministries of Defence, Interior and Justice

The security institutions provide external security and internal security for the country, often including the emergency preparedness and response command and control system. The Ministry of Health may provide the health contribution to this system and may be the primary source of ambulances, emergency medical teams, hospital emergency departments and hospital beds. The security ministries control the armed forces, the police, the intelligence services and the penal system. Personnel will either be formally uniformed with specific duties beyond general employment (eg, subject to military law rather than civilian law) or civilian employees. At least three ministries may provide health services for defined security populations.

The Ministry of Defence, through the Armed Forces, is usually responsible for protecting the integrity of the country from external threats. Most armed forces have an integral medical service that both maintains the health of armed forces personnel and can deploy in support of the armed forces.^12^ The beneficiaries of the military medical system will include military personnel, and may also include their families, veterans and civilian employees.^13^ Some military systems also treat civilians either on a private basis or as state-based funding. Military medical systems are often an integral part of the country’s emergency preparedness and response system as it is the only health system that is organised for field deployment and relatively uncommitted to the local civilian community.^14^

The Ministry of Interior is usually responsible for internal security through the management of police services. Police services may be split by different functions including border security, local policing, national response and paramilitary population control. In some countries, the police services have their own medical system like the Armed Forces. This may be necessary if the security environment is such that security personnel are not safe under medical care in the public health system.^15^

The Ministry of Justice usually controls the judicial and penal system. This will include responsibility for the health and welfare of prisoners. This is often a neglected component of a health system, although prisoners may have health needs (especially in mental health) that exceed the general population. These unique health needs and settings should be considered as part of a country’s public health system.^16^

There may be other state providers of health services. Delegated governance might place responsibility for health-related social services on regional governments or local authorities. This might include residential care for physically and mentally disabled citizens, and rehabilitation services. Large state employers might also provide occupational and curative health services for their workforce; examples include the railway sector^17^ or extractive industries.^18^

### The independent sector

The model separates the independent sector into those providers that raise charges for services for financial gain (commercial and not-for-profit providers) and those that provide subsidised services free or at nominal cost (the NGO and charity sector, including social enterprises). The commercial group covers those healthcare providers that operate to make a profit for shareholders. This includes private hospitals, diagnostic centres and other clinical services. This segment of the market represents an expanding component of health systems in many low-income and middle-income countries.^19^ The not-for-profit group covers healthcare providers that operate independently of government but are not profit generating for shareholders. Examples include occupational health services provided for employees of private companies, non-government insurance or mutually funded services, personally owned practices (including pharmacies) and traditional healers. The final category is the IA, NGO and charity sector. This can be divided into indigenous organisations and international organisations. The international group can also be divided into large multinational charitable IAs and NGOs that operate alongside the international development assistance funders and smaller charities that rely on private donations. Some multinational movements, such as the International Federation of Red Cross and Red Crescent Societies, have national societies that operate in most nations.

## Conclusions

This paper builds on previously published work and, through further analysis, testing and debate, the original framework has been refined to be valid as a generic tool for DHE. It provides a comprehensive description of the concepts and thinking behind this holistic framework that captures the full breadth of national government and non-government health service providers. In addition to the state-provided services for public/population health and curative care, other state actors include government ministries such as Higher Education, Defence, and Interior. The actors in the non-government sector includes commercial and private providers alongside NGOs and charities.

We offer this model as a conceptual framework that can inform the development of DHE tools including collaboration between DHE and wider civilian global health systems strengthening activities. The model allows an analysis of the interdependencies across health services providers in order to identify opportunities for collaboration or deconfliction in DHE beyond solely the Ministry of Defence. We intend to test the validity of this model through cross-disciplinary case studies of specific country health systems and to develop a method to illustrate the relative contribution of each provider to the whole system. The model may also help in understanding how patients navigate health systems to meet their health needs, including consideration of seeking health services from outside their country.^20^
